# Real-world analysis of pharmacological treatments to prevent relapse after electroconvulsive therapy for major depressive disorder: A nationwide cohort study

**DOI:** 10.1038/s41398-025-03746-0

**Published:** 2025-11-18

**Authors:** Alexander Kronsell, Axel Nordenskjöld, Robert Bodén, Ellenor Mittendorfer-Rutz, Johan Reutfors, Marios Rossides, Mikael Tiger

**Affiliations:** 1 Division of Insurance Medicine, Department of Clinical Neuroscience, Karolinska Institutet, Stockholm, Sweden; 2 Faculty of medicine and health, Örebro University, Örebro, Sweden; 3 Department of Medical Sciences, Uppsala University, Uppsala, Sweden; 4 Department of Medicine Solna, Karolinska Institutet, Stockholm, Sweden; 5 Institute of Environmental Medicine, Karolinska Institutet, Stockholm, Sweden; 6 Department of Respiratory Medicine and Allergy, Karolinska University Hospital, Stockholm, Sweden; 7 Centre for Psychiatry Research, Department of Clinical Neuroscience, Karolinska Institutet & Stockholm Health Care Services, Region Stockholm, Sweden

**Keywords:** Depression, Clinical pharmacology

## Abstract

During the first year following electroconvulsive therapy (ECT) for major depressive disorder (MDD) there is a large risk of relapse. Previous studies have shown favourable results for lithium after ECT for MDD. However, lithium is infrequently prescribed after ECT. While some evidence exists for other pharmacological strategies such as TCAs and TCA-lithium combinations, comparative data remain limited. The aim of this study was to explore pharmacological treatments after ECT for MDD and analyse their association with relapse following response to ECT for MDD. We hypothesized that lithium would be associated with a lower risk of relapse. We conducted a nationwide cohort study using data from Swedish registers. Patients 18 years or older with MDD who received ECT 2013-2019 and responded distinctly to ECT were followed for a year. Specified drugs dispensed up to four weeks after the ECT series were considered the exposure. Relapse was defined as psychiatric hospitalisation, renewed ECT, intentional self-harm, or death by suicide. Adjusted hazard ratios (aHR) and 95% confidence intervals (CI) were estimated using Cox models controlled for several potential confounders. The study population included 2 858 patients with distinct response to ECT. The most common psychiatric drugs dispensed after ECT were antipsychotics (39.7%), mirtazapine (38.0%), and selective serotonin reuptake inhibitors (SSRI) (35.9%). There was a statistically non-significant lower risk of relapse associated with lithium (aHR 0.86, 95% CI 0.69-1.07, p = 0.17). Antipsychotics were associated with a greater risk of relapse (aHR 1.17, 95% CI 1.05-1.31, p = 0.006). For other pharmacological treatments there were no associations with risk of relapse.

## Introduction

Electroconvulsive therapy (ECT) is an effective treatment for major depressive disorder (MDD) with response rates typically ranging from 50 to 70%. [1–3] However, after an initial treatment course, previous studies have reported relapse rates of 40% during the first six months, even with pharmacological therapy for relapse prevention. [4] Among patients receiving placebo after ECT, relapse-rates have been reported much higher, up to 84%. [5] There is no consensus on the optimal pharmacological treatment following response to ECT for MDD. [4, 6, 7] The most common strategies to prevent relapse after a successful ECT series include prescription of antidepressants, lithium, atypical antipsychotics, or continuation of ECT. For patients in remission, continuation of ECT is an effective strategy to prevent MDD relapse. [8] According to a randomized controlled trial, among patients with a combination of pharmacotherapy and continuation ECT, only 32% relapsed within one year, compared to 61% of those who received pharmacotherapy alone. [9]

Among patients with MDD who received ECT as their primary treatment, a previous small randomized study of relapse prevention after ECT showed results in favour of lithium in monotherapy compared to placebo. [10] In addition, another randomized trial showed that a combination of lithium and nortriptyline was superior to treatment with nortriptyline alone, with relapse rates of 84% for placebo, 60% for nortriptyline alone, and 39% for the combination therapy. [5] A third randomized study compared nortriptyline with venlafaxine, both in combination with lithium, and observed no difference in relapse rate, with high relapse rates despite pharmacological continuation therapy. [11] However, in a large register-based study, treatment with antidepressants after ECT was superior to no antidepressant, and lithium superior to no lithium, but the combination was not superior to lithium alone for preventing readmission. Dispensation of lithium, but not antidepressants, was associated with reduced risk of suicide. [12]

Among patients with MDD who did not receive ECT as their primary treatment, adding lithium to antidepressant treatment reduced depressive symptoms more than placebo and lithium treatment was associated with a lower risk of suicide in some studies. [13, 14] In an observational study, monotherapy with lithium was associated with a lower risk of relapse in patients with MDD compared to other pharmacological treatments. [15] Augmentation of acute antidepressant effect has been demonstrated with second generation antipsychotics(SGA). [16] However, regarding antipsychotics for maintenance treatment for MDD the results have been inconsistent. In one study olanzapine was shown to be effective in preventing relapse among patients with psychotic depression, however, other studies have shown no benefit with this treatment. [17, 18]

Despite some findings in favour of lithium, only 9% of patients were prescribed lithium after ECT in a previous study. [12] Real-life evidence is needed to test the effectiveness of lithium as maintenance treatment after ECT for MDD. [19] In addition, comparative data on other pharmacological strategies post-ECT remain limited. Our aim was to explore prescription patterns after ECT for MDD and analyse their association with relapse defined as psychiatric rehospitalization, renewed ECT, intentional self-harm, or suicide, following response to ECT for MDD. We hypothesized that lithium, whether used alone or in combination with other agents, would be associated with a lower risk of relapse.

## Materials and methods

### Study design

This is a nationwide observational study using data from national Swedish registers. Data regarding ECT procedures and outcomes were gathered from the Swedish National Quality register for ECT (Q-ECT). Data for the Q-ECT are gathered prospectively. During 2013 the Q-ECT had an inclusion rate of 85% and during 2019 93%. [20, 21] Data from the Q-ECT have previously been validated and shown up to 95% accordance when compared to patient records. [22] Data on comorbidities and pharmacotherapy were gathered from the National Patient Register and the National Prescribed Drug Register, respectively. A study protocol with included variables, outcomes and definitions was pre-registered. [23] This study was approved by the regional ethical review board in Uppsala, Sweden, and the Swedish Ethical Review Authority. No informed consent was required for this register-based study using anonymized data. The report of this study adhered to the STROBE guidelines for cohort studies. [24]

### Study population inclusion

All patients 18 years or older who were treated with ECT for MDD and included in the Q-ECT 2013-2019 were considered for inclusion. The following diagnoses, according to the International Classification of Diseases, Swedish version 10 (ICD-10), were included: F32.1 Moderate depressive episode, F32.2 Severe depressive episode without psychotic symptoms, F32.3 Severe depressive episode with psychotic symptoms, F33.1 Recurrent depressive disorder, current episode moderate, F33.2 Recurrent depressive disorder, current episode severe without psychotic symptoms and F33.3 Recurrent depressive disorder, current episode severe with psychotic symptoms. Only patients receiving an index series, defined as two or more treatments per week, of ECT were included, patients with continuation or maintenance treatments were not considered for inclusion. For our primary analysis, only patients with distinct response to treatment were included. Distinct response was defined as a score of 1 (1=very much improved) on the Clinical Global Impressions – Improvement scale (CGI-I). [25] In a secondary analysis, we included all patients that responded to treatment. This was defined as a CGI-I score of 1 (very much improved) or 2 (much improved). The rationale for using CGI-I = 1 in the primary analysis was to define a population most closely approximating clinical remission. This decision was made a priori, based on the assumption that relapse prevention strategies are most relevant and interpretable among patients with the most distinct treatment response. If a patient received several ECT series during the study period, the first series meeting the criteria of response was used.

### Exclusion

Patients with at least one visit listing an ICD-coded diagnosis in the National Patient Register of schizophrenia, schizoaffective disorder, psychosis, bipolar disorder, or epilepsy within 5 years before ECT were excluded (see Supplement for ICD-10 codes).

### Exposure

Exposure was defined as prescriptions dispensed during the last week of the ECT-series, and up to 4 weeks after the ECT series end, of the following drugs or drug categories: selective serotonin reuptake inhibitors (SSRI), serotonin–norepinephrine reuptake inhibitors (SNRI), mirtazapine, tricyclic antidepressants (TCA), other antidepressants (agomelatine, bupropion, mianserin, and vortioxetine), antipsychotics, lamotrigine, and lithium. Patients with no filled prescriptions during the 4-week period post-ECT but who were dispensed at least one of the drugs within 4 weeks before the start of ECT were considered exposed to the same drugs post-ECT. Data on dispensed prescriptions was gathered from the National Prescribed Drug Register and is recorded using Anatomical Therapeutic Chemical (ATC) codes (see supplementary material for specific ATC-codes). To prevent immortal time bias, patients were considered exposed only from the date of their first dispensation within the exposure window. If an outcome occurred before any such dispensation, the patient was classified as unexposed for that episode.

### Primary outcome and measurements

The primary outcome was relapse defined as a composite outcome of suicide, intentional self-harm, psychiatric hospitalisation or renewed ECT within a year after response to ECT. Suicide was defined using ICD-coded data from the National Cause of Death Register including unknown unnatural causes of death. Intentional self-harm was defined as any hospital admission with a primary or secondary discharge diagnosis of intentional self-harm. The following ICD-10 codes were used to define completed and attempted suicide: X60-X84 and Y10-Y34. In this study, the term intentional self-harm refers to hospital admissions for intentional self-harm or self-inflicted injuries of undetermined intent, as defined by ICD-10 codes X60–X84 and Y10–Y34. We acknowledge that these codes may include events with varying levels of suicidal intent. Psychiatric rehospitalisation was specified as any psychiatric rehospitalisation for any cause for a minimum of one calendar day, which was gathered from the National Patient Register. Renewed ECT was defined as a minimum of one ECT session administered as an additional series, based on data from the Q-ECT.

### Self-rating scales

The Swedish self-rated version of the Montgomery-Åsberg Depression Rating Scale (MADRS-S) was available in the Q-ECT register for a subset of patients. [26] The scale includes nine items rated from 0 to 6, covering core symptoms of depression. Although not used as an outcome measure in the present study, MADRS-S data were used for descriptive purposes in a subsample.

### Comorbidities

Data on psychiatric comorbidities and substance use disorders was gathered from the National Patient Register. A comorbidity (from the groups: anxiety disorders, personality disorders, and substance use disorders) was present if at least one ICD-10 code was registered within 3 years before the first ECT. Considering that ICD codes are recorded in the register at the end of a hospital stay, ICD codes registered within one week after completing treatment course or at discharge after the current ECT-series were also included. See supplementary material for a full list of ICD-10 codes.

### Statistical analysis

An intention-to-treat approach was used where switching or discontinuation of medications were not considered. Individuals were followed up from the end of the ECT series until the onset of the primary composite outcome (see above), death from other cause than suicide, or until a year from the end of the ECT series, whichever occurred first. Hazard ratios (HR) and 95% confidence intervals (CI) were estimated using multivariable Cox proportional hazards models associated with exposure (yes/no) to any of the drugs or drug categories defined above (see “Exposure”). An initial model was adjusted for age (continuous), sex, birth country (Nordic, non-Nordic), and education ( ≤ 9, 10–12, ≥13 years, missing). Our primary model was further adjusted for a number of different classes of psychiatric drugs dispensed within 2 years before start of ECT (continuous), number of psychiatric hospitalizations within five years before ECT (continuous), history of substance abuse (yes, no), history of psychiatric comorbidity (yes, no), psychotic symptoms during the current depressive episode (yes, no), and concomitant benzodiazepine use (yes, no), medication switch before and after the current ECT series. The proportional hazards assumption for the primary model was tested and the assumption was met (p = 0.32).

In the primary analysis, we excluded individuals (ECT-series) where CGI-I was missing. We conducted a sensitivity analysis to check the robustness of our findings in the presence of missing CGI-I using two extreme scenarios. In the first, all missing CGI-I was imputed with “1” indicating distinct response whereas in the second missing CGI-I was imputed as “3” indicating complete non-response.

Data management and analyses were conducted using SAS software version 9.4 (SAS Institute Inc., Cary, NC, USA).

### Missing data

Due to the mandatory nature of the inpatient portion of the National Patient Register, the National Prescribed Drug register and the National Cause of Death Register, we had complete data. However, a total of 2 726 (23.8%) patients of our initial patient selection had missing data on CGI-I. For further analysis of missing data, see sensitivity analysis.

## Results

### Study sample and exclusions

**Fig. 1:**
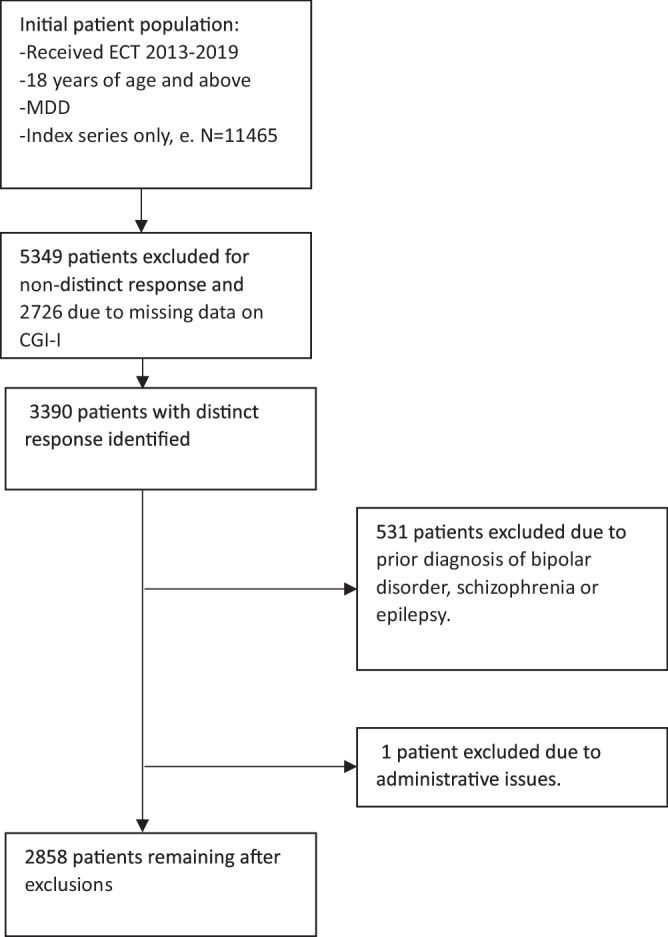
Flow-chart of study population. Illustrates each step of inclusion and exclusion for the study population and the size of the study population for each step of the primary analysis. ECT electroconvulsive therapy, MDD major depressive disorder, CGI-I clinical global impressions–improvement scale.

We found 11 465 patients meeting our initial eligibility criteria. For our primary analysis, 5349(46.7%) patients were excluded due to indistinct response, 2726(23.8%) due to missing data on CGI-I; 3 390 patients remained. An additional 531(4.6%) patients were excluded due to a prior diagnosis of bipolar disorder, schizophrenia, schizoaffective disorder, or epilepsy. Our final analysis sample included 2 858 patients with distinct response (Fig. 1). Using the same inclusion and exclusion criteria for our secondary analysis, we identified 6 760 responders, defined as individuals with a CGI-I score of 1 or 2.

### Sociodemographic and clinical characteristics

Table 1 shows clinical and sociodemographic characteristics for the two separate analysis, distinct responders and all responders. The average age among distinct responders was 59.9 (SD 17.4). More than half (57%) of patients among distinct responders were female and 37% had psychotic symptoms during their current depressive episode.

**Table 1:** Social demographics and clinical characteristics.

	Distinct Responders (CGI-I = 1), n = 2858	All Responders (CGI-I = 1 or 2), n = 6760
**Age, mean (SD)**	59.9 (17.4)	57.2 (18.1)
**Sex, n (%)**		
Male	1225 (42.9)	2892 (42.8)
Female	1633 (57.1)	3868 (57.2)
**Birth country, n (%)**		
Nordic	2624 (91.8)	6111 (90.4)
Non-Nordic	234 (8.2)	649 (9.6)
**Education, years, n (%)**		
≤9	615 (21.5)	1471 (21.8)
10–12	1246 (43.6)	2997 (44.3)
≥13	923 (32.3)	2076 (30.7)
Missing	74 (2.6)	216 (3.2)
Number of different medication categories 2 years before ECT, mean (SD)*	2.0 (1.7)	2.3 (1.8)
**Care type during ECT treatment, n (%)**		
Inpatient care	2326 (93.0)	5317 (91.6)
Outpatient care	177 (7.0)	489 (8.4)
**Number of hospitalizations within five years before ECT, mean (SD)**	1.3 (3.3)	1.4 (3.5)
**Psychiatric comorbidity**		
Substance abuse disorder, n (%)	267 (9.3)	789 (11.7)
Anxiety disorders, n (%)	192 (6.7)	583 (8.6)
Personality disorders, n (%)	97 (3.4)	369 (5.5)
**Psychotic symptoms during current depressive episode, n (%)**	1057 (37.0)	1903 (28.2)
**Concomitant benzodiazepine use, n (%)**	688 (24.1)	1765 (26.1)
**Medications dispensed 3 months before ECT**†**, n (%)**		
Lithium	24 (0.8)	53 (0.8)
SSRI	497 (17.4)	1156 (17.1)
SNRI	204 (7.1)	567 (8.4)
TCA	86 (3.0)	207 (3.1)
Mirtazapine	385 (13.5)	901 (13.3)
Another antidepressant	154 (5.4)	439 (6.5)
Antipsychotic	207 (7.2)	547 (8.1)
Lamotrigine	37 (1.3)	114 (1.7)
No medication	1648 (57.7)	3776 (55.9)

Describes characteristics for our primary analysis (distinct responders) and a secondary analysis (all responders).

*=Using the same category definitions as those utilized for our exposure.

†= Categories are not mutually exclusive and thus do not add up to 100%.

*ECT* electroconvulsive therapy, *SD* standard deviation, *CGI-I* clinical global impression–improvement scale, *SSRI* selective serotonin reuptake inhibitors, *SNRI* serotonin–norepinephrine reuptake inhibitors, *TCA* tricyclic antidepressants.

### Post-ECT prescription patterns

Prescription patterns of post-ECT maintenance medications are presented in Table 2. Among patients with distinct response, the three most common categories of psychiatric drugs dispensed were antipsychotics (39.7%), mirtazapine (38.0%) and SSRIs (35.9%). The most commonly prescribed antipsychotic drug was olanzapine (18.7%) and the most common SSRI was sertraline (14.8%).

**Table 2:** Distribution of prescriptions filled post ECT.

	Distinct Responders (CGI-I = 1), n = 2858	All Responders (CGI-I = 1 or 2), n = 6760
**SSRI, n (%)**	1027 (35.9)	2340 (34.6)
Sertraline	422 (14.8)	889 (13.2)
Escitalopram	358 (12.5)	856 (12.7)
Citalopram	134 (4.7)	305 (4.5)
Fluoxetine	74 (2.6)	182 (2.7)
Paroxetine	38 (1.3)	105 (1.6)
**SNRI, n (%)**	808 (28.3)	1863 (27.6)
Venlafaxine	519 (18.2)	1186 (17.5)
Duloxetine	289 (10.1)	677 (10.1)
**TCA, n (%)**	139 (4.9)	401 (5.9)
Clomipramine	100 (3.5)	260 (3.9)
Amitriptyline	25 (0.9)	106 (1.6)
Nortriptyline	14 (0.5)	35 (0.5)
**Mirtazapine, n (%)**	1086 (38.0)	2378 (35.2)
**Other Antidepressants, n (%)**	248 (8.7)	717 (10.6)
Bupropion	107 (3.7)	321 (4.8)
Vortioxetine	50 (1.8)	152 (2.3)
Mianserin	48 (1.7)	121 (1.8)
Agomelatine	36 (1.3)	97 (1.4)
**Antipsychotics, n (%)**	1134 (39.7)	2649 (39.2)
Olanzapine	535 (18.7)	1097 (16.2)
Quetiapine	275 (9.6)	718 (10.6)
Risperidone	150 (5.3)	324 (4.8)
Aripiprazole	61 (2.1)	151 (2.2)
Levomepromazine	45 (1.6)	154 (2.3)
Haloperidol	29 (1.0)	83 (1.2)
Flupentixol	11 (0.4)	35 (0.5)
Chlorprothixene	7 (0.2)	28 (0.4)
Clozapine	3 ( < 0.1)	17 (0.3)
**Lamotrigine, n (%)**	85 (3.0)	271 (4.0)
**Lithium, n (%)**	183 (6.4)	420 (6.2)
**Switch or add on of medication post ECT %**	2765 (96.8)	6498 (96.1)
**No dispensed medication four weeks post-ECT**	193 (6.8)	541 (8.0)

The above table describes the distribution of prescriptions filled from the pre-defined categories and the distribution of the individual drugs and their corresponding ATC-code within each category.Drugs prescribed to a total of 20 or less patients are not individually listed in the table but accounted for in the total count for each category.

*ECT* electroconvulsive therapy, *SD* standard deviation, *CGI-I* clinical global impressions – improvement scale, *SSRI* selective serotonin reuptake inhibitors, *SNRI* serotonin–norepinephrine reuptake inhibitors, *TCA* tricyclic antidepressants.

### Main findings

Main findings on the association of drug exposure and relapse after ECT are presented in Table 3. Among distinct responders, 52.3% relapsed during the first year after treatment. Among patients with distinct response that relapsed, 61.8% were hospitalized, 34.0% received a new series of ECT, 3.3% presented with intentional self-harm, and 0.9% died by suicide. Among all responders, 54.4% of patients relapsed during the first year. Among distinct responders, patients who filled a prescription for antipsychotics after ECT had a 17% increased risk of relapse compared to those who did not (HR 1.17, 95% CI 1.05-1.31, p = 0.006), after adjusting for exposure to other study medications and confounders. Lithium was associated with a non-significantly reduced risk of relapse (HR 0.86, 95% CI 0.69-1.07, p = 0.17). In a secondary analysis of all responders, patients who filled a prescription of antipsychotics had an increased risk of relapse (HR 1.11, 95% CI 1.03-1.19, p = 0.005). There were no other statistically significant associations between medications prescribed after ECT and an increased, or decreased, risk of relapse.

**Table 3:** Results from Cox proportional hazards model.

Distinct responders CGI-I = 1	N	Outcome n (%)	HR*(95% CI), initial model	HR*(95% CI), primary model	P-value, primary model
Lithium	183	90 (49.2)	0.90 (0.73–1.12)	0.86 (0.69–1.07)	0.17
SSRI	1027	524 (51.0)	0.94 (0.82–1.07)	0.91 (0.80–1.04)	0.18
SNRI	808	426 (52.7)	0.97 (0.84–1.11)	0.89 (0.78–1.02)	0.10
TCA	139	75 (54.0)	1.03 (0.80–1.32)	0.88 (0.69–1.13)	0.32
Mirtazapine	1086	547 (50.4)	0.95 (0.84–1.07)	0.93 (0.83–1.05)	0.22
Other antidepressants	248	156 (62.9)	1.30 (1.09–1.55)	1.08 (0.90–1.29)	0.43
Antipsychotic	1134	642 (56.6)	1.19 (1.07–1.32)	1.17 (1.05–1.31)	0.006
Lamotrigine	85	58 (68.2)	1.58 (1.21–2.06)	1.30 (1.00–1.71)	0.051
**All responders CGI-I** = **1 or 2**					
Lithium	420	211 (50.2)	0.91 (0.79–1.05)	0.88 (0.77–1.02)	0.09
SSRI	2340	1204 (51.5)	0.99 (0.91–1.08)	0.99 (0.91–1.07)	0.73
SNRI	1863	1020 (54.8)	1.08 (0.99–1.18)	1.03 (0.94–1.13)	0.50
TCA	401	234 (58.4)	1.18 (1.02–1.35)	1.05 (0.91–1.22)	0.47
Mirtazapine	2378	1230 (51.7)	1.01 (0.94–1.09)	1.02 (0.94–1.10)	0.63
Other antidepressants	717	422 (58.9)	1.17 (1.05–1.31)	1.03 (0.92–1.15)	0.63
Antipsychotic	2649	1499 (56.6)	1.15 (1.08–1.24)	1.11 (1.03–1.19)	0.005
Lamotrigine	271	166 (61.3)	1.14 (0.97–1.33)	0.98 (0.84–1.15)	0.84

This table shows hazard ratios and their corresponding 95% confidence intervals. In addition, for our primary model p-values have been calculated.

*HRs and 95% CIs from Cox proportional hazards models. Multivariable analysis comparing dispensation vs. non-dispensation of each medication controlling for other dispensed medications. A HR above 1.0 indicates dispensation is associated with an increased risk of relapse compared to no dispensation of that drug. The initial model is adjusted for age (continuous), sex (female, male), birth country (Nordic, non-Nordic), and education ( ≤ 9, 10¬–12, ≥13 years, missing). The primary model is further adjusted for number of medication categories dispensed within 2 years before start of ECT (continuous), number of hospitalizations within five years before ECT (continuous), history of substance abuse (yes, no), history of psychiatric comorbidity (yes, no), psychotic symptoms during depressive episode (yes, no), and concomitant benzodiazepine use (yes, no), medication switch before and after ECT (yes, no).

*ECT* electroconvulsive therapy, *HR* hazard ratio, *CI* confidence interval, *CGI-I* clinical global impressions – improvement scale, *SSRI* selective serotonin reuptake inhibitors, *SNRI* serotonin–norepinephrine reuptake inhibitors, *TCA* tricyclic antidepressants.

### Post-hoc analyses

As a part of a post-hoc analysis we saw that among distinct responders, 55.4% of patients with psychotic symptoms were dispensed antipsychotics and 30.4% of patients without psychotic symptoms were dispensed antipsychotics after ECT. Among patients with distinct response, 62.4% had available data on MADRS-S post treatment. The mean MADRS-S score among distinct responders’ post-index treatment was 8 (SD 7), where remission is defined a score of 10 or less. [26] Both those prescribed an antipsychotic drug and those who were not had the same average score. There was no statistically significant difference between these groups (p-value: 0.83). In an additional post-hoc analysis, we examined the proportion of patients who refilled a prescription for the same drug within three months of the initial dispensation. Among patients with lithium 86.9% of patients filled a new prescription within three months and among patients with antipsychotics 77.7% of patients re-filled their prescription. Data on all categories can be found in the supplementary material.

### Sensitivity analysis

Patients with missing data on CGI-I were compared to our primary group of patients, distinct responders. Patients with missing data on CGI-I were on average younger than distinct responders (56.5 vs. 59.9 years, P < 0.001). No significant differences were observed in gender distribution. The group with missing data on CGI-I had a higher proportion of patients with psychiatric comorbidities (22.7 vs. 12.9%, P < 0.001) and substance use disorders (13.0 vs. 9.3%, P < 0.001) compared to distinct responders. Patients with missing data on CGI-I had a lower frequency of psychotic symptoms during their depressive episode (19.4 vs 37.0%, p < 0.001).

Due to the missing data regarding CGI-I, a sensitivity analysis for distinct responders with two scenarios was carried out. In the first, all patients with missing CGI-I score were assumed to be distinct responders, while in the second they were considered as non-responders. In these scenarios, we observed the following statistically significant results; in the first scenario, lithium was associated with a lower risk of relapse (HR 0.84, 95% CI 0.71-0.99) and antipsychotics were associated with a higher risk of relapse (HR 1.34, 95% CI 1.23-1.46). In the second scenario, lithium was associated with a non-significant reduced risk of relapse(HR 0.91, 95% CI 0.78-1.06) and antipsychotics were associated with a higher risk of relapse (HR 1.28, 95% CI 1.17-1.39). Full results of these two models can be found in the supplementary material. In an additional sensitivity analysis, we considered all medications dispensed two months before ECT and one month after ECT as the exposure, thus widening the definition. Findings were similar in this model. Full results from this model can be found in the supplementary material.

## Discussion

### Main findings and comparison with findings from other studies

The aim of this study was to examine real-world prescription patterns following ECT for major depressive disorder and to explore how these patterns relate to the risk of relapse. Although there was a non-significant association between lithium and a reduced risk of relapse, we were unable to corroborate our hypothesis that lithium would be associated with a lower risk of relapse following ECT for MDD. By contrast, we found an association between dispensation of antipsychotics and an increased risk of relapse. For other medications, we observed no statistically significant associations with risk of relapse.

Among distinct responders, 52.3% relapsed within the first year after receiving treatment with ECT for MDD. The high rate of relapse post ECT is in line with previous studies, where 51% of patients relapsed within one year. [4] A previous study showed an association between lithium and lower risk of relapse in a similar patient population. [12] However, we could not replicate this finding in our study. In our study there was a trend for association between lithium and a lower risk of relapse, although this was not statistically significant. The study by Brus et al. looked at all patients that received ECT, not just responders, and that study included a total of 7 350 patients, a larger study population than ours. Thus, the non-significance of the reduced risk of relapse with lithium dispensation after ECT in our study could possibly be attributed to a lack of power. Our primary patient population, distinct responders, had a higher frequency of psychotic symptoms and a lower frequency of psychiatric comorbidity compared to the previous study’s population. In addition, the outcome was defined as suicide or hospital readmission, while our study also included intentional self-harm or renewed ECT. When considering patients who did not respond to ECT and their subsequently prescribed medications, the research question shifts from relapse prevention to treatment of ECT-resistant depression, and the latter question was not considered for our study.

In our material we found that a large portion of patients with MDD were prescribed antipsychotics after treatment. There are previous studies based on Swedish registry data examining drugs prescribed post ECT, [12] but large-scale prescription patterns following ECT in other countries are largely unknown. A previous study suggested that olanzapine may be effective for relapse prevention in patients with psychotic depression; however, the evidence is not definitive, and this potential benefit was not specifically examined in patients who received ECT as their primary treatment. [17] In our material, we observed that responders who were dispensed an antipsychotic post ECT were more likely to relapse. Increased risk of relapse after ECT in patients dispensed antipsychotics has been reported in previous studies but have then been attributed to confounding by indication and it has been suggested that patients who receive antipsychotics post ECT have a more severe disease and residual symptoms. [12, 27] Altogether, there is currently no support for the use of antipsychotics after ECT for MDD. In addition, there is a risk of side-effects of certain second-generation antipsychotics, such as weight gain and diabetes mellitus. [28]

The association of an increased risk of relapse for patients prescribed antipsychotics was persistent in both our primary and secondary analysis, in initial and final models, and three scenarios in our sensitivity analysis. In addition, our model was adjusted for several factors that account for disease severity, such as previous number of hospital admissions for psychiatric reasons, previous number of prescribed antidepressant drugs (indicating treatment resistance) and psychotic features during the current episode. Despite these adjustments, residual confounding and confounding by indication still cannot be ruled out. Prescribing decisions may also have been influenced by clinical impressions not captured in structured data, such as personality traits or perceived relapse risk. Additionally, considering that 37% of distinct responders were prescribed an antipsychotic post ECT it would be difficult to argue that these patients are exceptional, considering that they make up a large portion of the study population. Furthermore, patients who were prescribed an antipsychotic post ECT did not have a higher MADRS-S score post treatment. However, MADRS-S data were only available for a subset of patients, and the scale may not fully capture differences in symptom profiles. If confounding by indication was a particular issue with our model, one could argue that similar patterns would have been seen for patients who were prescribed lithium or TCA, as these are typically reserved for MDD patients where other treatments have failed. [29] However, lithium and TCAs are typically prescribed as continuous treatments, whereas antipsychotics may often be used for intermittent use. As such, intermittent use of antipsychotics may serve as a proxy for acute severity, and this could contribute to the observed association with relapse through confounding by indication.

### Strengths

Our study is the first to investigate the effectiveness of a wide range of psychiatric medications in preventing relapse among patients who have responded to ECT in a real-world setting. The real-world setting provides an opportunity to evaluate treatment strategies in a less controlled environment, enabling the inclusion of patients who would not qualify for a controlled study, in a large number of patients. Our models were adjusted for a wide range of highly detailed data from validated Swedish registries. Another strength of the study is the large, nationwide sample of patients, which increases the generalizability of the findings.

### Limitations

This was an observational study using real-world data, and any associations observed should be interpreted with caution. Even though our models are adjusted for several variables, there is still a risk of confounding by indication. A large portion of patients had missing data on response (CGI-I) and the group with missing data on CGI-I were different than patients with available data regarding age, psychiatric comorbidities and substance use disorder. As these patients were more frequently excluded due to missing data, this could cause selection bias. However, a sensitivity analysis was conducted for two extreme scenarios regarding patients with missing data on CGI-I. In these two scenarios, our main findings were not altered. In addition, the CGI-I scale, is a relatively coarse measure, that is not specific for change in depressive symptoms. An additional limitation is that there is no data on the indication for the medications dispensed, as an example, some patients might have been prescribed mirtazapine or quetiapine for sleeping disorders and not as a relapse prevention strategy post ECT. Furthermore, we were unable to determine whether prescribed medications, particularly antipsychotics, were intended for regular or intermittent use, which may have influenced the observed associations. Another concern is the high proportion of included patients who were prescribed SSRIs or mirtazapine after ECT. These treatments have limited evidence as maintenance strategies post-ECT and are generally considered less effective than tricyclic antidepressants in patients hospitalized for MDD. [30] This prescribing pattern may have impacted the observed relapse rates in our study. Finally, our definition of relapse, which is based on hospitalization, new ECT series, suicide or intentional self-harm, may miss cases of clinical deterioration managed in outpatient settings, such as patients experiencing increased depressive symptoms without hospitalization or further ECT. A potential limitation of our statistical model is that, due to the four-week exposure period, the exposure could in some cases occur after the outcome. To address this limitation, we conducted an additional sensitivity analysis, which yielded similar results (See Table 4 in supplementary). An additional potential limitation is that patients with lithium prescriptions prior to ECT were not excluded, which could introduce prevalent user bias through depletion of susceptibles.

### Conclusions and clinical implications

The risk of relapse is high even after distinct response to ECT for MDD, despite pharmacological maintenance treatment. Even though there was a trend for association between lithium and lower risk of relapse, we could not corroborate our primary hypothesis. Antipsychotics were commonly prescribed after ECT for MDD and dispensation of antipsychotics was associated with a higher risk of relapse. Currently, there is no evidence supporting the use of antipsychotics as a relapse prevention strategy among patients who have received ECT for MDD. Thus, initiation of antipsychotic drugs after ECT for MDD should be evaluated in further studies before routine implementation in clinical health care.

## Supplementary information

Supplemental material

## Data Availability

The data that support the findings of this study are not publicly available due to privacy concerns and the sensitive nature of patient information. Access to the data may be granted upon request to the Swedish National Quality Register for ECT, in accordance with applicable regulations.
