# Supplementary material for: Real-world analysis of pharmacological treatments to prevent relapse after electroconvulsive therapy for major depressive disorder: A nationwide cohort study
Source: Transl Psychiatry. 2025 Nov 18;15:514. doi: 10.1038/s41398-025-03746-0 (PMC12669661; doi:10.1038/s41398-025-03746-0)
Supplement: Supplementary file 1 — Supplemental material [file 41398_2025_3746_MOESM1_ESM.docx]

**Supplementary material**

Drug exposure 1

Psychiatric comorbidities 2

Sensitivity analysis 3

Re-filled prescriptions 4

**Supplementary material - Exposure**

Exposure was defined as prescriptions filled during the last week of the ECT-series and up to 4 weeks after the last session in the series of the following drugs or category of drugs, irrespective of date of prescription from the below categories. Below ATC-codes for each category is listed.

**SSRI:** N06AB**

**SNRI:** N06AX16, N06AX21

**TCA:** N06AA**

**Mirtazapine:** N06AX11

**Other antidepressants:** N06AX03, N06AX12, N06AX22, N06AX26, N06AG02, N06AF04, N06AX02

**Antipsychotics:** N05A***, except lithium N05AN01.

**Lamotrigine:** N03AX09

**Lithium:** N05AN01

**Combinations with lithium (primary analysis). Not mutually exclusive.**

**Table 1**

|  | **Distinct Responders (CGI-I=1), n=2858** | **All Responders (CGI-I= 1 or 2), n=6760** |
| --- | --- | --- |
| **Combinations with Lithium, n (%)** |  |  |
| Lithium alone | 33 (1.2) | 64 (1.0) |
| Lithium & SSRI | 39 (1.4) | 84 (1.2) |
| Lithium & SNRI | 57 (2.0) | 131 (1.9) |
| Lithium & TCA | 24 (0.8) | 49 (0.7) |
| Lithium & Mirtazapine | 37 (1.3) | 108 (1.6) |
| Lithium & Other antidepressant | 15 (0.5) | 42 (0.6) |
| Lithium & Antipsychotic | 78 (2.7) | 187 (2.8) |
| Lithium & Lamotrigine | 8 (0.3) | 23 (0.3) |

**Supplementary material – Psychiatric comorbidities**

**Psychiatric comorbidities – ICD-10**

A comorbidity was considered to be present if the corresponding ICD-10 code was registered within 3 years before the first date of ECT. Considering that ICD-codes are recorded in the register at the end of a hospital stay, ICD-codes registered within one week after concluded treatment or at discharge after the current ECT-series were also included.

**Psychiatric comorbidity:**

F41.0-F41.1(anxiety disorders)
F60.0-F60.9(personality disorders)

**Substance use disorder:**

F10.0-F10.9
F11.0-F11.9
F12.0-F12.9
F13.0-F13.9
F14.0-F14.9
F15.0-F15.9
F16.0-F16.9
F18.0-F18.9
F19.0-F19.9

**Sensitivity analysis**

**Scenario 1 and 2 – missing data on CGI-I**

For scenario 1 all patients with missing data on CGI-I are considered distinct responders (CGI=1). For scenario 2 all patients with missing data on CGI-I are considered non-responders (CGI-I=3 or more)

**Table 2**

| **Exposure drug** | **Missing CGI-I is considered as distinct response (=1)  HR*(95% CI), primary model** | **Missing CGI-I is considered as non-response (>3)**  **HR*(95% CI), primary model** |
| --- | --- | --- |
| Lithium | 0.84 (0.71– 0.99) | 0.91 (0.78– 1.06) |
| SSRI | 0.96 (0.87– 1.06) | 1.07 (0.97– 1.19) |
| SNRI | 0.93 (0.84– 1.03) | 1.02 (0.92– 1.13) |
| TCA | 0.99 (0.84– 1.18) | 1.12 (0.97– 1.30) |
| Mirtazapine | 1.02 (0.93– 1.12) | 1.05 (0.95– 1.15) |
| Another antidepressant | 1.01 (0.88– 1.15) | 0.95 (0.84– 1.08) |
| Antipsychotic | 1.34 (1.23– 1.46) | 1.28 (1.17– 1.39) |
| Lamotrigine | 1.16 (0.98– 1.38) | 0.99 (0.84– 1.17) |
|  |  |  |

**Scenario 3 – Broader definition of exposure**

All medications prescribed two months before ECT and up to 1 month after are considered as exposure.

**Table 3**

| **Distinct responders CGI-I=1** | **N** | **Outcome n (%)** | **HR*(95% CI), Model 1** | **HR*(95% CI), primary model** | **P-value, primary model** |
| --- | --- | --- | --- | --- | --- |
| Lithium | 205 | 102 (49.8) | 0.89 (0.72– 1.09) | 0.87 (0.71– 1.07) | 0.18 |
| SSRI | 1449 | 726 (50.1) | 0.91 (0.81– 1.02) | 0.90 (0.80– 1.02) | 0.09 |
| SNRI | 977 | 534 (54.7) | 1.04 (0.92– 1.17) | 0.95 (0.84– 1.08) | 0.45 |
| TCA | 203 | 113 (55.7) | 1.04 (0.85– 1.28) | 0.92 (0.75– 1.12) | 0.40 |
| Mirtazapine | 1404 | 712 (50.7) | 0.95 (0.85– 1.07) | 0.93 (0.83– 1.05) | 0.24 |
| Another antidepressant | 377 | 230 (61.0) | 1.25 (1.08– 1.44) | 1.03 (0.88– 1.20) | 0.72 |
| Antipsychotic | 1321 | 742 (56.2) | 1.17 (1.06– 1.30) | 1.13 (1.01– 1.26) | 0.04 |
| Lamotrigine | 117 | 77 (65.8) | 1.39 (1.10– 1.75) | 1.11 (0.88– 1.41) | 0.38 |
| *HRs and 95% CIs from Cox proportional hazards models. Multivariable analysis comparing dispensation vs. non-dispensation of each medication controlling for other dispensed medications. Model 1 is adjusted for age (continuous), sex (female, male), birth country (Nordic, non-Nordic), and education (≤9, 10¬–12, ≥13 years, missing). The primary model is further adjusted for number of medication categories dispensed within 2 years before start of ECT (continuous), number of hospitalizations within five years before ECT (continuous), history of substance abuse (yes, no), history of psychiatric comorbidity (yes, no), psychotic symptoms during depressive episode(yes, no), and concomitant benzodiazepine use (yes, no), medication switch before and after ECT (yes, no). | | | | | |
| ECT=Electroconvulsive Therapy, HR=Hazard Ratio, CI=Confidence Interval, CGI-I=Clinical Global Impressions – Improvement scale, SSRI=Selective Serotonin Reuptake Inhibitors, SNRI=Serotonin–norepinephrine reuptake inhibitors, TCA=Tricyclic Antidepressants | | | | | |

**Sensitivity analysis: Start of follow-up at a month after the end of the ECT series (individuals had to not have had the outcome within the first month from the end of the series).

Table 4**

| **Distinct responders CGI-I=1**  **(N=2237)** | **N** | **Outcome n (%)** | **HR*(95% CI), initial model** | **HR*(95% CI), primary model** | **P-value, primary model** |
| --- | --- | --- | --- | --- | --- |
| Lithium | 131 | 38 (29.0) | 0.65 (0.46, 0.90) | 0.60 (0.43, 0.83) | 0.002 |
| SSRI | 814 | 311 (38.2) | 0.94 (0.80, 1.12) | 0.92 (0.78, 1.09) | 0.36 |
| SNRI | 628 | 246 (39.2) | 0.97 (0.81, 1.17) | 0.89 (0.74, 1.07) | 0.22 |
| TCA | 99 | 35 (35.4) | 0.86 (0.60, 1.23) | 0.71 (0.50, 1.02) | 0.06 |
| Mirtazapine | 867 | 328 (37.8) | 0.91 (0.78, 1.06) | 0.89 (0.76, 1.03) | 0.12 |
| Other antidepressants | 188 | 96 (51.1) | 1.48 (1.18, 1.87) | 1.21 (0.96, 1.53) | 0.11 |
| Antipsychotic | 871 | 379 (43.5) | 1.25 (1.09, 1.44) | 1.20 (1.04, 1.40) | 0.01 |
| Lamotrigine | 58 | 31 (43.5) | 1.67 (1.16, 2.40) | 1.31 (0.91, 1.89) | 0.15 |
| No medication | 157 | 55 (35.0) | 0.94 (0.68, 1.30) | 0.81 (0.54, 1.22) | 0.32 |
| **All responders CGI-I= 1 or 2**  **(N=5223)** |  |  |  |  |  |
| Lithium | 316 | 108 (34.2) | 0.80 (0.66, 0.97) | 0.75 (0.62, 0.92) | 0.005 |
| SSRI | 1829 | 696 (38.1) | 0.97 (0.87, 1.08) | 0.96 (0.86, 1.07) | 0.44 |
| SNRI | 1413 | 571 (40.4) | 1.05 (0.94, 1.19) | 1.00 (0.89, 1.12) | 0.97 |
| TCA | 292 | 125 (42.8) | 1.12 (0.92, 1.35) | 0.97 (0.80, 1.18) | 0.75 |
| Mirtazapine | 1878 | 733 (39.0) | 1.01 (0.91, 1.12) | 1.02 (0.92, 1.12) | 0.73 |
| Other antidepressants | 551 | 256 (46.5) | 1.31 (1.14, 1.51) | 1.10 (0.96, 1.27) | 0.18 |
| Antipsychotic | 2048 | 902 (44.0) | 1.28 (1.16, 1.40) | 1.21 (1.10, 1.33) | <0.001 |
| Lamotrigine | 205 | 100 (48.8) | 1.29 (1.05, 1.58) | 1.06 (0.86, 1.30) | 0.57 |
| No medication | 408 | 170 (41.7) | 1.26 (1.04, 1.53) | 1.11 (0.87, 1.41) | 0.41 |
| *HRs and 95% CIs from Cox proportional hazards models. Multivariable analysis comparing dispensation vs. non-dispensation of each medication controlling for other dispensed medications. A HR above 1.0 indicates dispensation is associated with an increased risk of relapse compared to no dispensation of that drug. The initial model is adjusted for age (continuous), sex (female, male), birth country (Nordic, non-Nordic), and education (≤9, 10¬–12, ≥13 years, missing). The primary model is further adjusted for number of medication categories dispensed within 2 years before start of ECT (continuous), number of hospitalizations within five years before ECT (continuous), history of substance abuse (yes, no), history of psychiatric comorbidity (yes, no), psychotic symptoms during depressive episode (yes, no), and concomitant benzodiazepine use (yes, no), medication switch before and after ECT (yes, no). | | | | | |

**Renewed prescriptions filled within 3 months**

The below table shows the proportion of patients who re-fill their prescription of the same drug within 3 months of the initial filled prescription. Data is grouped for each category of drugs.

**Table 5**

| **Distinct responders CGI-I=1** | **N** | **Refill, n (%)** |
| --- | --- | --- |
| Lithium | 183 | 159 (86.9) |
| SSRI | 1027 | 722 (70.3) |
| SNRI | 808 | 624 (77.2) |
| TCA | 139 | 109 (78.4) |
| Mirtazapine | 1086 | 720 (66.3) |
| Other antidepressants | 248 | 176 (71.0) |
| Antipsychotics | 1134 | 881 (77.7) |
| Lamotrigine | 85 | 62 (72.9) |
